# Differential Effect of HDAC3 on Cytoplasmic and Nuclear Huntingtin Aggregates

**DOI:** 10.1371/journal.pone.0111277

**Published:** 2014-11-07

**Authors:** Tatsuo Mano, Takayoshi Suzuki, Shoji Tsuji, Atsushi Iwata

**Affiliations:** 1 Department of Neurology, Graduate School of Medicine, The University of Tokyo, Tokyo, Japan; 2 Department of Graduate School of Medical Science, Kyoto Prefectural University of Medicine, Kyoto, Japan; 3 Japan Science and Technology Agency, Precursory Research for Embryonic Science and Technology (PRESTO), Saitama, Japan; National Center of Neurology and Psychiatry, Japan

## Abstract

Histone deacetylases (HDACs) are potential therapeutic targets of polyglutamine (pQ) diseases including Huntington’s disease (HD) that may function to correct aberrant transcriptional deactivation caused by mutant pQ proteins. HDAC3 is a unique class 1 HDAC found in both the cytoplasm and in the nucleus. However, the precise functions of HDAC3 in the two cellular compartments are only vaguely known. HDAC3 directly binds to huntingtin (Htt) with short pQ and this interaction is important for suppressing neurotoxicity induced by HDAC3. With long pQ Htt, the interaction with HDAC3 is inhibited, and this supposedly promotes neuronal death, indicating that HDAC3 would be a good therapeutic target for HD. However, the knockout of one HDAC3 allele did not show any efficacy in reducing neurodegenerative symptoms in a mouse model of HD. Therefore, the role of HDAC3 in the pathogenesis of HD has yet to be fully elucidated. We attempted to resolve this issue by focusing on the different roles of HDAC3 on cytoplasmic and nuclear Htt aggregates. In addition to supporting the previous findings, we found that HDAC3 preferentially binds to nuclear Htt over cytoplasmic ones. Specific HDAC3 inhibitors increased the total amount of Htt aggregates by increasing the amount of nuclear aggregates. Both cytoplasmic and nuclear Htt aggregates were able to suppress endogenous HDAC3 activity, which led to decreased nuclear proteasome activity. Therefore, we concluded that Htt aggregates impair nuclear proteasome activity through the inhibition of HDAC3. Our findings provide new insights regarding cross-compartment proteasome regulation.

## Introduction

In polyglutamine (pQ) diseases, the gene transcription machinery required for proper neuronal function is impaired, and this may result from the sequestration of essential proteins for transcription [1]–[4] and/or the abnormal hypo-acetylation of the genome [5]. The up-regulation of transcription by histone deacetylase (HDAC) inhibitors was shown to be an effective treatment in a fly model of pQ disease [6]. Since then, multiple studies have shown that HDAC inhibitors ameliorate symptoms and pathology in various models of Huntington’s disease (HD), one of the major pQ diseases [7]–[11]. However, broad-spectrum HDAC inhibitors used in these studies have multiple targets and should therefore be avoided for therapeutic purposes. Indeed, considering that the inhibition of HDAC6 has a negative effect on pQ degradation [12], caution is needed when interpreting data from these broad-spectrum inhibitor studies. Moreover, these broad-spectrum inhibitors are not suitable for use as actual medicines to be administered to human subjects because of the potential for unwanted side effects.

There are four classes of HDACs and among them, class I or IIa HDACs have been previously suggested as therapeutic targets for pQ diseases [13]. Classes I and IIa each contain four HDACs, and in order to narrow down the therapeutic target, various studies using specific inhibitors or genetic ablation strategies have been performed. The results seem to consistently show that inhibition of HDAC1, 2, or 4 leads to some improvement [11], [14]–[16] and inhibition of HDAC6 or 7 has no effect, at least at doses that can be administered without any negative effects in animal models [17], [18]. The results for HDAC3 inhibition are mixed. While one study using a specific HDAC3 inhibitor showed phenotypic improvement in a fly model [16], another study showed no effect in the offspring of crossbred HDAC3 knockout and HD model mice [19]. One possibility for this discrepancy is that the HDAC3 inhibitor used in the first study was not specific enough and that the observed improvement was a result of the inhibition of other HDACs. In addition, it is possible that the genetic ablation in the second study did not achieve enough inhibition since the study was performed using hemi-zygote HDAC3 knockout mice because the full knockout resulted in embryonic lethality.

Another possible cause of this discrepancy is that unlike HDAC1 or 2, which only functions at the nucleus, HDAC3 can shuttle between the cytoplasm and the nucleus where it can have different roles. Therefore, the effect of HDAC3 inhibition on HD models can depend on the balance of nuclear vs. cytoplasmic aggregates. In the case of pQ diseases, nuclear aggregates exhibit a far higher toxicity than cytoplasmic aggregates [20], [21] and there are cellular machineries that can only facilitate aggregate degradation in either the cytoplasm or in the nucleus [22], [23]. Inhibitors against proteins that shuttle between the cytoplasm and the nucleus might have a differential effect on aggregate degradation in different cellular compartments.

To overcome these issues, we utilized highly specific HDAC3 inhibitors made by a click chemistry-based combinatorial fragment assembly technique (Table S1) [24]. These HDAC3 inhibitors have an IC50 for HDAC3 that is at least 100-fold higher than that for other HDACs. By utilizing these reagents, we used cell lines that stably express pQ aggregates in different cellular compartments [23] to precisely analyze the role of HDAC3. Here, we show that these specific HDAC3 inhibitors affect cytoplasmic and nuclear huntingtin (Htt) aggregates differently. Moreover, the presence of intracellular aggregates also affected HDAC3 activity, indicating that HDAC3 could be an indirect regulator of proteasome function.

## Materials and Methods

### Cell culture and transfection of mammalian cells

HeLa and 293T cells were grown in 95% air and 5% CO_2_ at 37°C. Cells were transfected with plasmids using Lipofectamine 2000 (Life Technologies, Carlsbad, CA, USA) following the manufacturer’s protocol. The transfection efficiency was 60–75% for HeLa cells and >90% for 293T cells.

### Cell viability assay

Cells were incubated with CellTiter 96 Aqueous solution for an hour and absorbance at 490 nm was measured by the Spectra Max 384 Plus colorimetric plate reader (Molecular Devices, Sunnyvale, CA, USA).

### Filter trap assay

The filter trap assay was performed as previously described [12].

### HDAC3 constructs

HDAC3 cDNA was cloned from a cDNA library with oligonucleotide primers 5′ -CATGGCCAAGACCGTG- 3′ and 5′ -AAAGAAATTCCTTGGGACACA-3′. The HDAC3 knockdown construct was made with oligonucleotide primers, 5′- GATCCCCGATGCTGAACCATGCACCTTTCAAGAGAAGGTGCATGGTTCAGCATCTTTTTA-3′ and 5′- AGCTTAAAAAGATGCTGAACCATGCACCTTCTCTTGAAAGGTGCATGGTTCAGCATCGGG-3′ and was inserted into the pSuper vector (Oligoengine, Seattle, WA, USA). HDAC3 inactive mutants were PCR generated with oligonucleotide primers 5′ -TCGGGTGCTCTACATTGCCATTGCCATCCACCATGGTGA-3′ and 5′ -TCACCATGGTGGATGGCAATGGCAATGTAGAGCACCCGA-3′.

### HDAC3 inhibitors

Details about HDAC3 inhibitors T130, T247, and T326 were previously published [24]. Trichostatin A and suberoylanilide hydroxamic acid (SAHA) were purchased from Sigma Aldrich (St. Louis, MO, USA).

### HDAC activity assay

Pan-HDAC activity was assayed using the Flour-de-lys HDAC assay kit (Enzo Life Sciences, Farmingdale, NY, USA). The fluorometric assay was performed using the Spectramax Gemini XS (Molecular Devices) with an excitation wavelength of 360 nm and emission at 460 nm. HDAC3 activity was assayed using the HDAC3 activity assay kit (Sigma Aldrich) with excitation at 380 nm and emission at 500 nm.

### Image quantitation

Western blot images were obtained using a LAS 3000 Mini (Fujifilm, Tokyo, Japan). Digital images were analyzed by Multi Gauge software (Fujifilm).

### Immunoprecipitation and GST pull down analysis

To prepare lysates for immunoprecipitation, cells were sonicated in 50 mM Tris, pH 7.5, 150 mM NaCl, 1% NP40, 1 mM ethylenediaminetetraacetic acid (EDTA), and Complete protease inhibitor cocktail (Roche, Basel, Switzerland) and centrifuged at 20,000×g for 15 min. Lysates were incubated with 1 µg of anti-FLAG M2 antibody immobilized agarose beads (Sigma) for 4 h at 4°C and washed for four times with lysis buffer and subjected to SDS-PAGE and Western blot analysis. For the glutathione S-transferase (GST) pull-down assay, cells were lysed in 20 mM HEPES, pH 7.5, 100 mM NaCl, 0.1% Triton X-100, 10% glycerol, and Complete protease inhibitor cocktail (Roche). GST or GST-HDAC3 (500 ng) was mixed with glutathione sepharose beads (Amersham Biosciences, Uppsala, Sweden) and incubated with the lysates for 2 h at 4°C. Beads were washed four times with the lysis buffer and subjected to SDS-PAGE and Western blot analysis.

### Microscopic imaging

Cells were fixed with 4% paraformaldehyde and a standard immunocytochemistry procedure was performed. Visualization of the primary anti-HDAC3 antibody (Imgenex, San Diego, CA, USA). was done with the Alexa 546 secondary antibodies (Life Technologies). Nucleus was visualized by Hoechst 33258 (Sigma Aldrich). Images were obtained using an Axioplan 2 fluorescent microscope and Axiocam HRc CCD camera system (Zeiss, Göttingen, Germany).

### Proteasome activity assay

Proteasome activity was measured using a 20S Proteasome Activity Assay Kit (Merck, Darmstadt, Germany) following the manufacturer’s protocol. The fluorometric assay was performed using a Spectramax Gemini XS (Molecular Devices) with an excitation wavelength of 380 nm and emission at 460 nm.

### Proteasome purification

Proteasomes were purified using the Proteasome purification kit (Enzo Life Sciences) following the manufacturer’s instructions.

### Quantitative PCR

Total RNA was extracted with TRIzol (Life Technologies) and cDNA was generated by ReverTra Ace qPCR RT Kit (Toyobo, Osaka, Japan). Quantitative PCR (qPCR) was performed with the HT-7900 system (Applied Biosystems, Foster City, CA, USA). For qPCR, the probe set Mr04097229_mr was used to measure EGFP mRNA, and HuGAPDH and HuACTB (Applied Biosystems) were used as internal controls.

### SDS-PAGE and western blot

Samples were incubated at 60°C in 4×LDS buffer (Life Technologies) for 15 min and subjected to SDS-PAGE with Mini-PROTEAN TGX gels (Bio-RAD, Hercules, CA, USA) and transferred to PVDF membrane with the Trans-Blot Turbo Blotting System and Trans-Blot Turbo Transfer Pack (Bio-RAD). For the primary antibodies, Anti-GFP (Roche), anti-20S proteasome antibody (Abcam, Cambridge, UK), anti-actin antibody (Millipore, Billerica, MA, USA), anti-FLAG antibody (Sigma Aldrich), anti-HDAC3 antibody (Abcam and Imgenex, San Diego, CA, USA), anti-GST antibody (Millipore), anti-HSP90 antibody (Millipore), anti-SP1 antibody (Millipore), anti-acetylated lysine antibody (Cell Signaling, Boston, MA, USA), anti-HSP70 antibody (StressMarq, Victoria, BC, Canada) were used.

### Stable cell lines

Stable HeLa cell lines expressing green fluorescent protein (GFP) fused to huntingtin exon-1 (Htt-ex1) with a nuclear export signal (NES), or nuclear localization signal (NLS) was previously published [23]. Cells with NES and CAG repeat lengths of 25, 47, and 72 were named E1, E2, E3, respectively, and cells with NLS were designated N1, N2, and N3, respectively. Cells without any localizing signals were named C1, C2, C3, and C4 with their CAG repeat lengths in ascending order (Table S2). The expressed protein sequence of Htt exon-1 (Htt-ex1) was “MATLEKLMKAFESLKSF(Q)_n_PPPPPPPPPPPQLPQPPPQAQPLLPQPQPPPPPPPPPPGPAVAEEPLHRP” which was followed by an EGFP sequence.

### Statistical analysis

Statistical analysis was performed using the GraphPad Prism 6 software (GraphPad, San Diego, CA, USA). The significance was tested with t-tests or ANOVA with Dunnett’s multiple comparisons.

### Subcellular fractionation

Cytoplasmic and nuclear fractions were extracted using NE-PER Nuclear and Cytoplasmic Extraction Kit (Thermo Scientific, Rockford, IL, USA).

## Results

### HDAC3-specific inhibitors affect the degradation of aggregation-prone Htt-ex1

**Figure 1:**
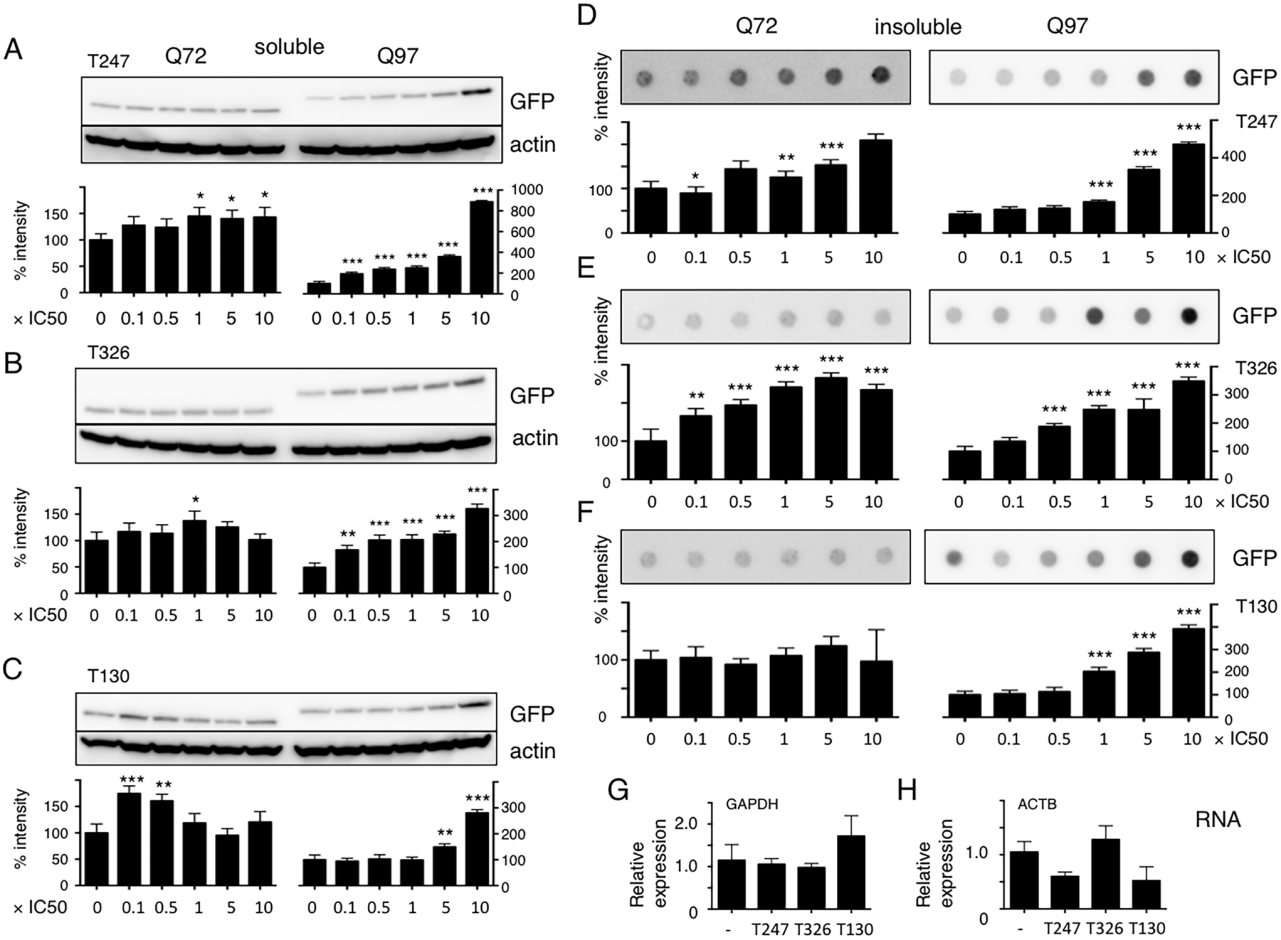
HDAC3 inhibitors increase both soluble and insoluble Htt-ex1s but prefer long Qs. **A–F:** Indicated amounts of HDAC3 inhibitors T247, T326, and T130 were added to C3 or C4 HeLa stable cell lines. The cells were harvested after 48 h of incubation and the fraction soluble in 1% Triton X-100 was subjected to western blot analysis (A–C). The insoluble fraction was subjected to a filter trap assay (D–E). Signals were detected by anti-GFP antibodies and chemiluminescence. Signal intensities were normalized to no inhibitors (DMSO only)  = 100. The band from an anti-actin blot is shown as a loading control. Panels A, D: T247, B, E: T326, C, F: T130. *P≤0.05, **P≤0.01, ***P≤0.001 vs. 0×IC50 by ANOVA with multiple comparisons. N = 3. **G, H:** HDAC3 inhibitors do not increase Htt-ex1 mRNA levels. Effect of HDAC3 inhibitors on Htt-ex1 expression levels were assayed by qPCR. G: internal control  =  GAPDH, H: internal control  =  ACTB. Expression level was normalized to no inhibitor = 1.0. There was no statistical significance by ANOVA with multiple comparisons. N = 3.

Generation of aggregated over soluble species is essential for Htt toxicity. To understand the effect of HDAC3 inhibition on the amount of Htt aggregates, we used stable HeLa cell lines that express Htt exon-1 (Htt-ex1) with various pQ lengths (Table S2) [23]. Three HDAC3 inhibitors had no effect on Htt-ex1 Q25 (C1 cells) and Htt-ex1 Q46 (C2 cells) (Figure S1), increased soluble Htt-ex1 Q72 to some extent, and significantly increased insoluble Htt-ex1 Q72 (C3 cells), and also significantly increased both soluble and insoluble Htt-ex1 Q97 (C4 cells) (Fig. 1A–F). None of the HDAC3 inhibitors showed a significant effect on Htt-ex1 mRNA levels (Fig. 1G, H); therefore, we concluded that HDAC3 inhibition affected the intracellular Htt-ex1 aggregate degradation pathway.

### HDAC3 activity reduces the amount of nuclear Htt-ex1 aggregates

**Figure 2:**
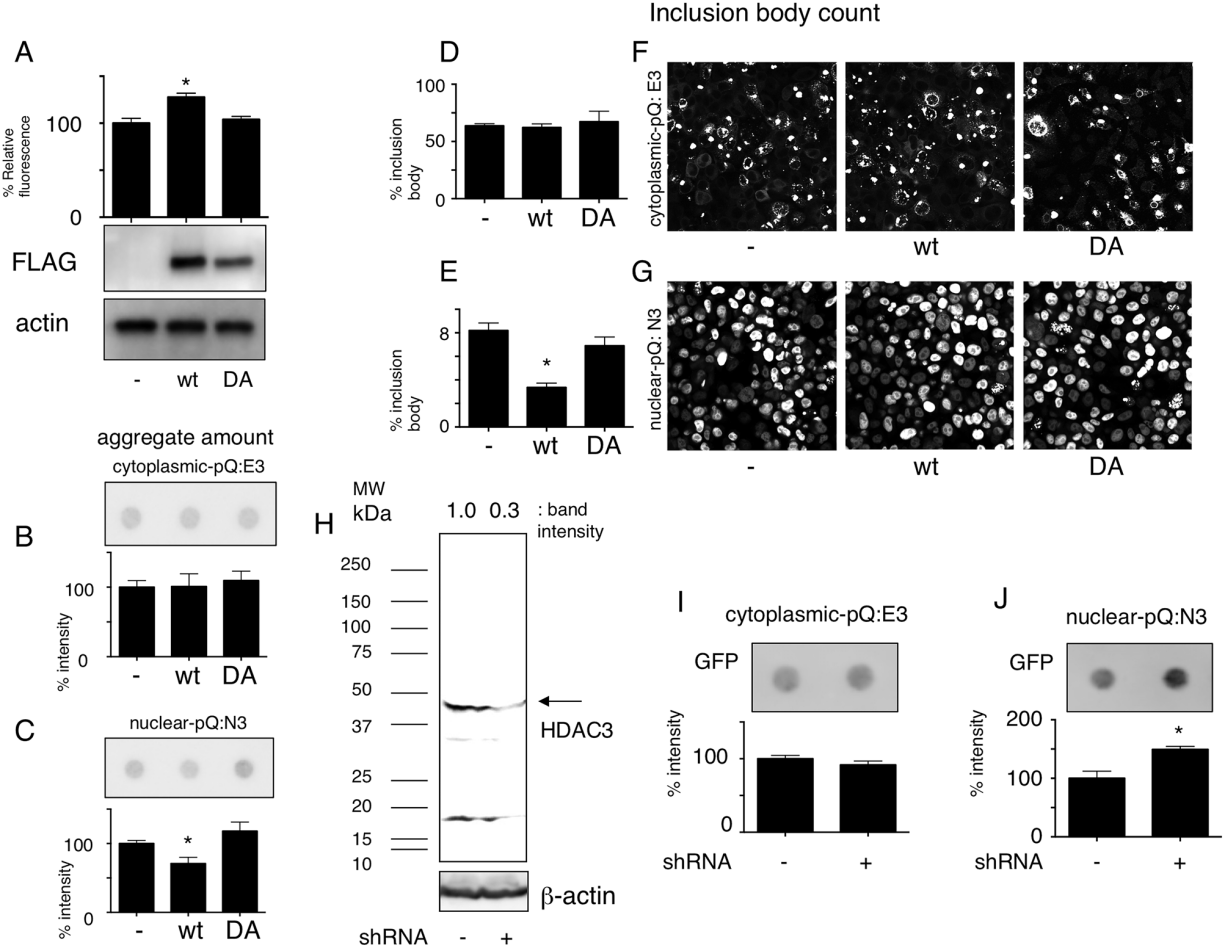
Effect of HDAC3 on cytoplasmic and nuclear Htt aggregates. **A:** Aspartate at the 166^th^ and 168^th^ amino acid of HDAC3 is crucial for its activity. An empty plasmid (–), FLAG tagged wild-type (wt), or D166A + D168A mutant (DA) of HDAC3 were overexpressed in 293T cells. After immunoprecipitation using anti-FLAG antibodies, pan-histone deacetylase activity was measured by fluorometric analysis. *P≤0.05 vs. empty plasmid by ANOVA and multiple comparisons. N = 3. Anti-FLAG and anti-actin western blots from cell lysates are shown below. **B–C:** HDAC3 overexpression reduces nuclear Htt-ex1 aggregates. Empty vector (–), FLAG-tagged wild-type or DA mutant HDAC3 were transfected to E3 and N3 cells. Amount of aggregate measured by filter trap assay are shown in B and C. *significant against – and DA by ANOVA and multiple comparisons. N = 3. **D–G**: Empty vector (–), FLAG-tagged wild-type or DA mutant HDAC3 were transfected to E3 and N3 cells. Cells harboring inclusion bodies are counted and their fraction in total cells was plotted in 2D and E. Representative GFP images of low powered magnification fields are shown in 2F and 2G. *significant against – and DA by ANOVA and multiple comparisons. **H:** HDAC3 shRNA reduces HDAC3 amount by 70%. Molecular weight markers are shown at the left side. **I–J:** HDAC3 knockdown increases nuclear aggregates. HDAC3 shRNA was transfected into E3 or N3 cells and the 1% TritonX-100 insoluble fraction was subjected to filter trap assay. *P = 0.0003 by *t*-test. N = 3.

To confirm that HDAC3 activity was important for the results of the previous experiments, and to see the effect of HDAC3 inhibition independently in the cytoplasm and the nucleus, we first generated plasmid constructs with either wild-type HDAC3 or a deacetylase activity-defective HDAC3 mutant. The key amino acids for HDAC3 activity were predicted to be the 166^th^ and 168^th^ aspartates; therefore, we mutated these amino acids to alanine, which successfully resulted in the loss of deacetylase activity (Fig. 2A). We then transfected these constructs into cell lines that stably express Htt-ex1 Q72 in the cytoplasm (E3 cells) or in the nucleus (N3 cells) and observed a significant decrease of biochemically (Fig. 2B, C) or microscopically (Fig. 2D–G) aggregated nuclear Htt-ex1 in accordance with HDAC3 activity. We also generated an HDAC3 knockdown construct that was able to reduce the amount of HDAC3 by 70% (Fig. 2H). We transfected these constructs into E3 and N3 cells and observed a significant increase of aggregated Htt-ex1 only in the N3 cells upon HDAC3 knockdown (Fig. 2I, J). These results show that HDAC3 activity negatively affected the amount of nuclear Htt-ex1 aggregates.

**Figure 3:**
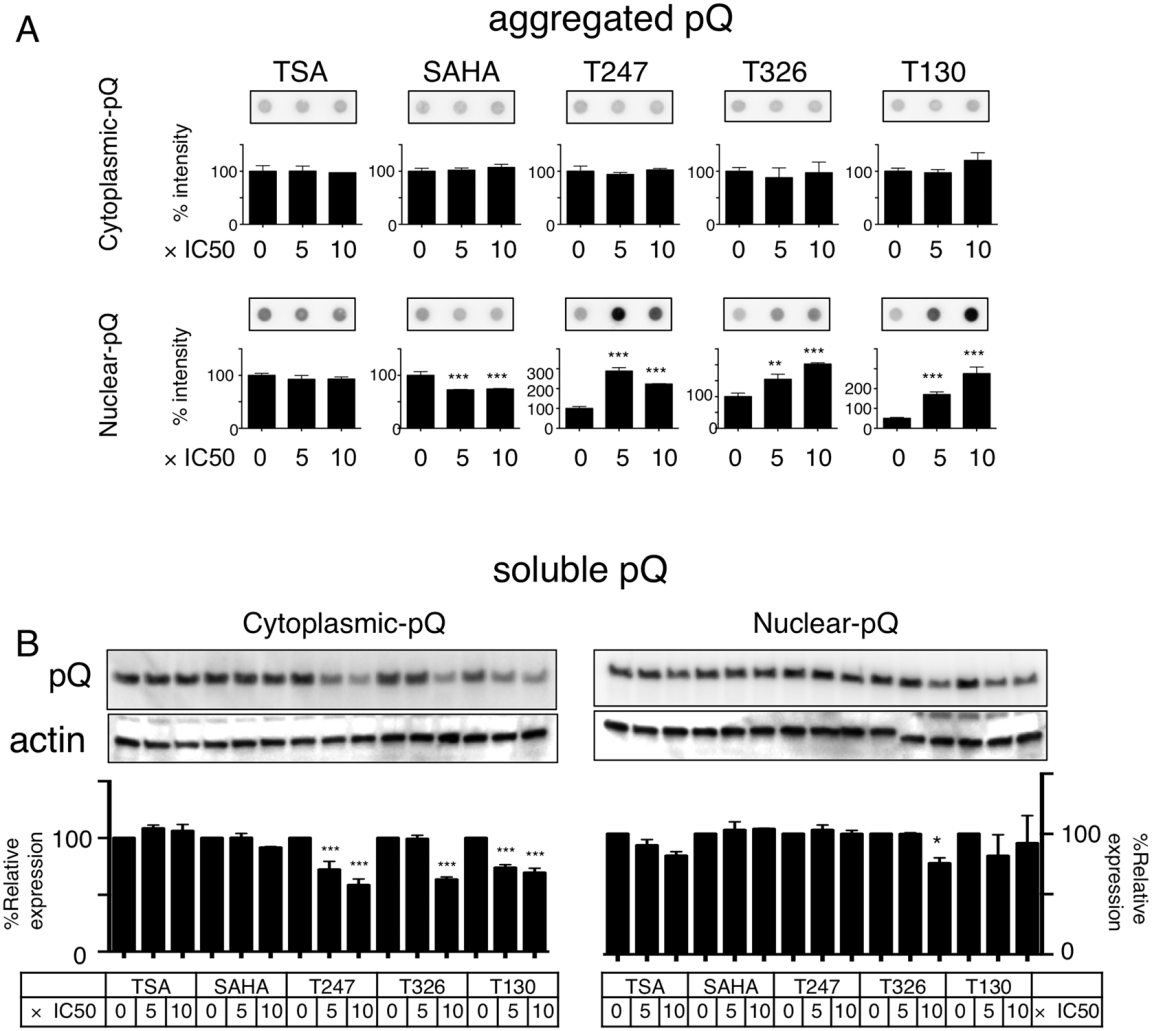
HDAC3 inhibitors have differential effects on cytoplasmic and nuclear Htt-ex1 aggregates. **A:** HDAC3 inhibitors increase aggregated nuclear Htt-ex1. For filter trap analysis, three independently made insoluble fractions were analyzed on one single membrane; thus, there are error bars shown for 0×IC50s. *P≤0.05, ***P≤0.001 vs. each 0×IC50 by ANOVA and multiple comparisons. N = 3. **B:** HDAC3 inhibitors reduce cytoplasmic soluble Htt-ex1s. The effect of various HDAC inhibitors on 1% TritonX-100 soluble cytoplasmic Htt-ex1s. Indicated amount of HDAC inhibitors were added to E3 (cytoplasmic) or N3 (nuclear) cells for 48 h. Quantitated band intensity was normalized to each band without HDAC inhibitors (0×IC50); thus, there are no error bars. **P≤0.01, ***P≤0.001 vs. each 0×IC50 by ANOVA with multiple comparisons. N = 3. Anti-actin blots are shown for loading control.

We then used HDAC3 specific inhibitors on the E3 and N3 cells. The results clearly showed that the HDAC3 inhibitors specifically increased the amount of nuclear Htt-ex1 aggregates and had no effect on cytoplasmic Htt-ex1 aggregates. Non-specific HDAC inhibitors TSA and SAHA had very little to no effect on the amount of either cytoplasmic or nuclear Htt-ex1 aggregates (Fig. 3A). HDAC3 inhibitors decreased soluble cytoplasmic Htt-ex1 (Fig. 3B).

### HDAC3 preferably binds to nuclear pQs

**Figure 4:**
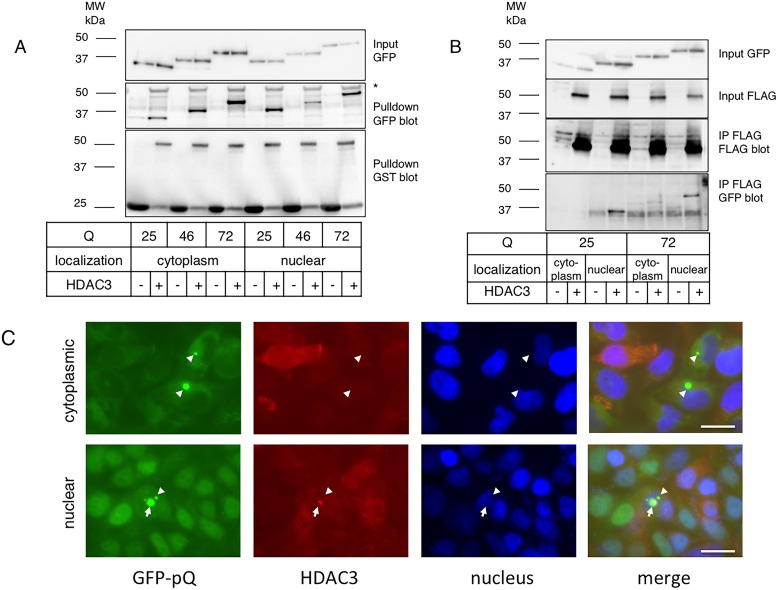
HDAC3 preferably binds to nuclear Htt with long Qs. **A:** GST-HDAC3 binds directly to either cytoplasmic or nuclear Htt-ex1. GST pull-down assay of E1, E2, E3 (cytoplasm), and N1, N2, N3 (nuclear) HeLa cell lysates is shown. Pulled-down fraction was analyzed by anti-GFP or GST antibodies. *Non-specific band. **B:** HDAC3 immunoprecipitates almost exclusively with nuclear Htt-ex1s. E1, E3 (cytoplasm), N1, and N3 (nuclear) HeLa cells were transfected with FLAG-tagged HDAC3 and those lysates were immunoprecipitated with anti-FLAG antibodies immobilized to protein G agarose beads. The pre-IP fraction and the IPed fraction were analyzed using anti-FLAG or anti-GFP antibodies. Molecular weight markers are shown on the left. **C:** HDAC3 associates exclusively with nuclear inclusion bodies. E3 or N3 cells were fixed and stained with anti-HDAC3 antibodies and visualized by Alexa 546 conjugated secondary antibodies. Arrowheads: inclusion bodies with no HDAC3 signals associated. Arrows: HDAC3 signal-associated inclusion bodies. Bar = 20 µm.

HDAC3 was previously reported to associate with pQ-containing proteins [25], [26]. We tested whether this association was a direct one using a GST pull-down assay. From sonicated lysates of E1, E2, E3, N1, N2, and N3 cells, GST-HDAC3 successfully pulled down both NES- and NLS-attached Htt-ex1s (Fig. 4A). We then performed immunoprecipitation using E1, E3, N1, and N3 cell lysates transfected with FLAG-tagged HDAC3. Interestingly, FLAG-tagged HDAC3 preferably co-immunoprecipitated with nuclear Htt-ex1s (Fig. 4B) and HDAC3 exhibited increased binding to 25Qs over 72Qs. This was confirmed with immunocytochemistry. Nuclear inclusion bodies in N3 cells displayed positive signals for endogenous HDAC3; however, cytoplasmic inclusion bodies from E3 cells were negative for HDAC3 (Fig. 4C). Thus, we concluded that HDAC3 binds to Htt-ex1s preferably inside the nucleus.

### HDAC3 inhibition caused by Htt-ex1 aggregates affects nuclear proteasome activity

**Figure 5:**
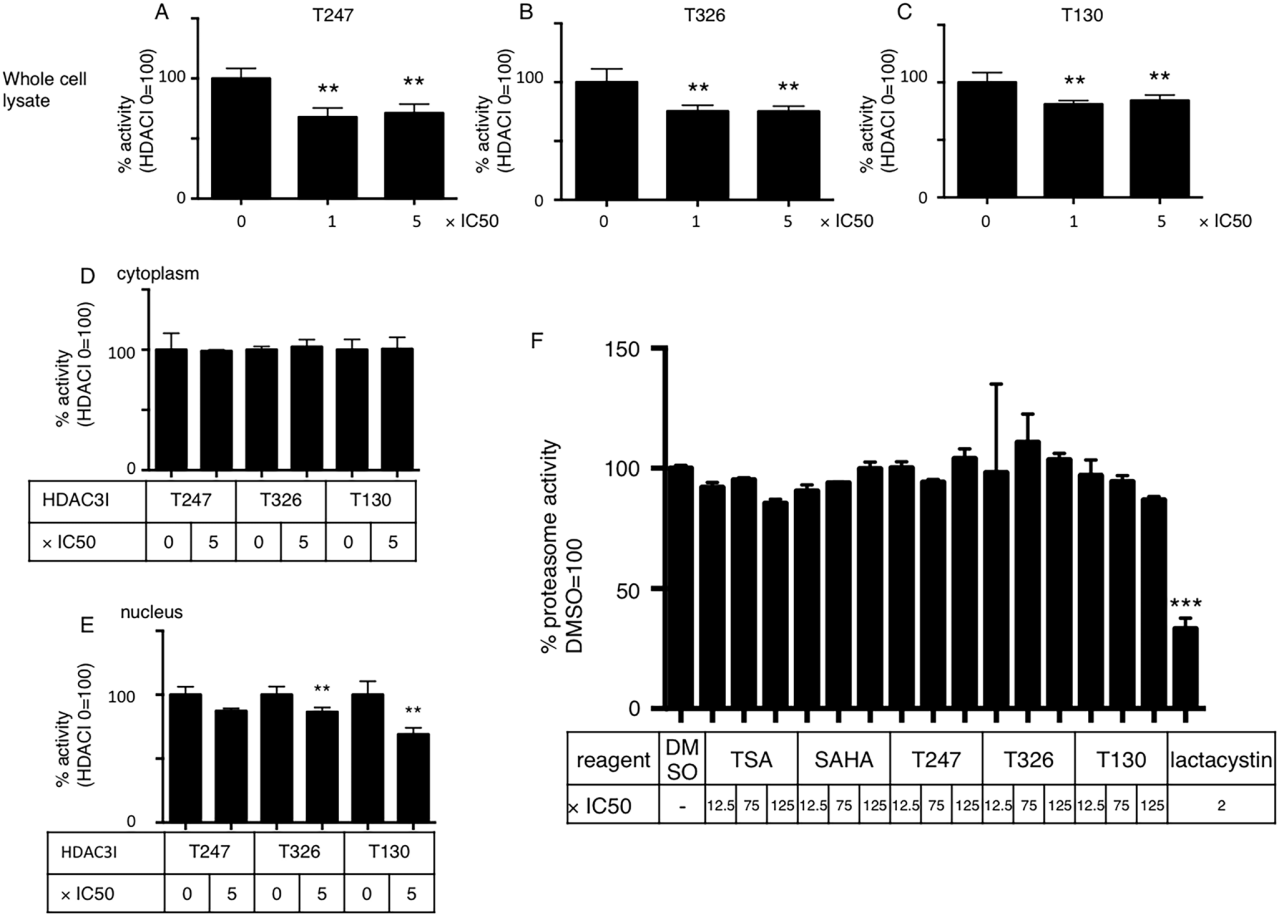
HDAC3 inhibitors affect proteasome activity. **A–C:** HDAC3 inhibitors inhibit proteasome activity. Three different HDAC3 inhibitors were added to HeLa cell cultures at the indicated concentrations. After 48 h of incubation, the proteasome activity of 5 µg total protein in a PBS lysate was measured using a fluorometric assay. A: T247, B: T326, C: T130. *P≤0.05 vs. each 0×IC50 by ANOVA with multiple comparisons. N = 3. **D, E:** HDAC3 inhibitors show a differential effect on cytoplasmic and nuclear proteasome activity. After incubating with the indicated amount of HDAC3 inhibitors for 48 h, cells were fractionated and the proteasome activity of 5 µg total protein from each fraction was independently measured. *P<0.05, **P<0.001 0×IC50 vs. 5×IC50 by *t*-tests N = 3. **F:** HDAC inhibitors have little or no direct proteasome inhibitory effect. Total protein (5 µg) from a HeLa cell PBS extract was subjected to the proteasome activity assay. During the incubation period for activity measurement, the indicated amount of HDAC inhibitors, or lactacystin as positive control, were added. Relative activity was shown with DMSO = 100%. ***P≤0.001 vs. DMSO by ANOVA with multiple comparisons. N = 3.

**Figure 6:**
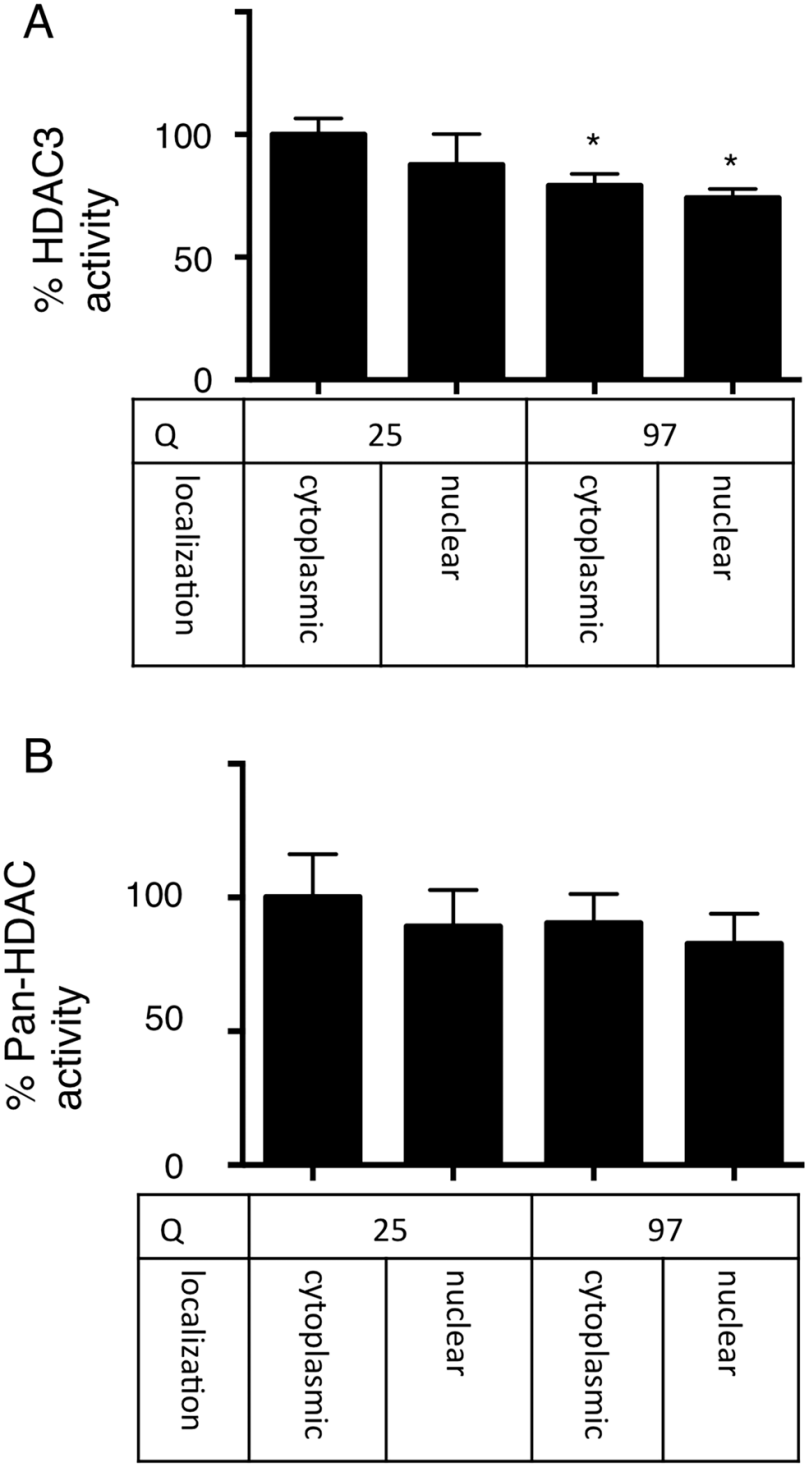
Htt aggregates inhibit HDAC3 activity. **A:** HDAC3 activity is suppressed upon either cytoplasmic or nuclear Htt-ex1 expression. After two days of transfection in 293T cells, the HDAC3 activity of cellular lysates was measured using a fluorescence-based assay. **B:** Htt-ex1 overexpression does not alter pan-HDAC activity. After two days of transfection in 293T cells, the pan-HDAC activity was measured using a fluorescence-based assay.

As shown in Figure 1, specific HDAC3 inhibitors increased Htt-ex1. It was either possible that HDAC3 inhibited its degradation or promoted its stability. Therefore we tested if HDAC3 inhibitors had any direct effect on the function of proteasomes, the major degradation machinery in the cell. We added HDAC3 inhibitors to HeLa cells and measured proteasome activity using a fluorometric assay. HDAC3 inhibitors impaired proteasome activity by 10–30%, even at a very low dose, with no further effect at higher doses (Fig. 5A–C). We then determined if these inhibitors had different effects on cytoplasmic and nuclear proteasome activity. As expected, HDAC3 inhibitors impaired nuclear proteasome activity but had no effect on cytoplasmic proteasomes (Fig. 5D, E). HDAC3 inhibitors did not directly inhibit proteasome activity (Fig. 5F). HDAC3 inhibitors did not affect the localization of the proteasome (Fig. S2), the acetylation level of Htt-ex1s or the proteasome (Fig. S3A, B, S4A). HDAC3 inhibitors did not affect the expression levels of HSP70, a chaperone that facilitates Htt-ex1 degradation by the proteasome (Fig. S4B). HDAC3 also does not bind to the proteasome and act as a scaffold between Htt-ex1 aggregates and the proteasome (Fig. S5). Thus, we speculated that proteasome function was impaired through an indirect pathway and hypothesized that the Htt-ex1 aggregates themselves affected HDAC3 activity. Therefore, we measured HDAC3 activity in the presence or absence of cellular aggregates. We showed that HDAC3 activity was suppressed by the overexpression of aggregate-prone Htt-ex1, regardless of its cellular localization (Fig. 6A). This effect was relatively specific to HDAC3 since pan-HDAC activity was not significantly affected by the same Htt-ex1 overexpression (Fig. 6B).

## Discussion

### Role of HDAC3 in pQ disease pathogenesis

The effect of HDAC3 inhibition in studies using pQ model animals has been controversial. While injection of an HDAC3 inhibitor into R62 mice seems to be effective in restoring the expression of genes that have been compromised by HD [16], the genetic knockdown of HDAC3 did not alter the phenotypic and pathological appearance of the same mice [19]. The latter study used HDAC3 hemizygote knockdowns that only achieved 10–20% reduction in the amount of HDAC3 at a protein level possibly being an insufficient inhibition. This discrepancy could also be due to specificity of the HDAC3 inhibitor used in the earlier study. Another possibility is that HDAC3 could have a particular function in HD pathogenesis, such that its inhibition might have multiple effects on the pathway and make the results difficult to interpret.

Then how is HDAC3 involved in HD pathogenesis? Previous studies have emphasized that HDAC3 itself is a neurotoxic protein that was neutralized by normal Htt when they are bound to each other. Since it prefers Htt with short Q as a binding partner (Figure 4B), unbound HDAC3 in the presence of long Q Htt exhibits its neurotoxicity [25] [27]. HDAC3 interacts with ataxin-7, another nuclear pQ protein, and stabilizes its post-translational modification [26]. HDAC3 is a class I HDAC that is abundantly expressed in the brain [28]. It is known to associate with and to be activated by a nuclear receptor co-repressor in order to control circadian metabolic physiology [29]. HDAC3 is also thought to have cytoplasmic function upon axonal injury [30], but its precise role is still unclear. The molecule has both a nuclear localization signal and a nuclear export signal [31], suggesting that it has different roles in the cytoplasm and the nucleus.

Our results indicate that HDAC3 inhibition could be beneficial in accelerating cytoplasmic Htt-ex1 pQ aggregation, but it inhibits the degradation of nuclear Htt-ex1 aggregates. In our study, we could not find an ideal dosage of HDAC3 inhibitors that would accelerate cytoplasmic aggregate degradation and not affect nuclear aggregate degradation, indicating that the therapeutic window for using HDAC3 inhibitors to treat HD could be very narrow, if it exists.

A previous report showed that HDAC inhibitors targeting HDAC1/3 prevent the formation of Htt aggregates in the brains of N171-82Q HD transgenic mice [32]. It is possible that this effect could be mediated by HDAC1 since acetylation of Htt can promote its autophagic clearance [33]. Thus the effect of HDAC3 could differ between cytoplasmic and nuclear aggregates.

### Indirect proteasome inhibition by Htt-ex1 aggregates could be linked to HDAC3

Our results clearly indicated that HDAC3 inhibitors impaired nuclear proteasome activity. Since direct incubation of the proteasome activity assay with HDAC3 inhibitors did not show any decrease in activity, this inhibition was determined to be an indirect effect of the inhibitors. Although there could be some non-specific background protease activity that was detected by our method, our results (Fig. 5F) show that this background activity could be negligible. We sought the mechanism of the inhibitory pathway and showed that it did not result from changes in the acetylation of the substrate Htt-ex1s or the proteasome itself. The localization of the proteasome was not affected by the inhibitors. In addition, we showed that HSP70, a chaperone that accelerates Htt degradation, was not affected by HDAC3 inhibitors. Thus, proteasome impairment was not a direct effect of HDAC3 inhibitors, but there was an indirect signaling pathway through HDAC3 to be discovered. We measured HDAC3 activity upon Htt-ex1 transfection and showed that both cytoplasmic and nuclear Htt-ex1 aggregates reduced endogenous HDAC3 activity.

In cellular models of HD, it has been reported that proteasome function has been impaired [34]. This inhibition is not a direct effect of the aggregates but rather an indirect effect where the full players are still unknown [35]. This inhibitory effect bi-directionally crosses the nuclear envelope [36], that is, nuclear aggregates inhibit cytoplasmic proteasome activity and vice versa. The mechanism of this phenomenon is unknown but our results suggest that at least nuclear proteasome function is impaired through the inhibition of HDAC3 by Htt-ex1s.

Our findings clearly demonstrate that HDAC3 inhibition is not a reasonable therapeutic target for HD. However, our results can lead to a better understanding of the regulation of proteasome function in different cellular compartments and provide new insight into proteasome inhibition by aggregated proteins.

## Supporting Information

Figure S1 **HDAC3 inhibitors do not have any effect on soluble Htt-ex1s.** C1 and C2 cells were incubated with the indicated amount of HDAC3 inhibitors for 48 h. The fractions soluble in 1% Triton X-100 from the filter trap assay were subjected to western blot analysis. There were no detectable filter trapped aggregates in these cells. Molecular weight markers are shown at the left side.
(TIF)

Figure S2 **The amount of 20S proteasome in each cellular compartment was not affected by HDAC3 inhibition HeLa cells were incubated with indicated amount of HDAC3 inhibitors for 48** **h, and nuclear and cytoplasmic fractions were extracted.** HSP90 and Sp1 blots are shown for the purity of the fractions. There was a slight increase of cytoplasmic HDAC3 and a slight decrease of nuclear HDAC3 upon addition of inhibitors. Molecular weight markers are shown at the left side.
(TIF)

Figure S3 **A, B:** HDAC3 inhibitor does not alter the acetylation level of Htt-ex1s. E3 (NES) or N3 (NLS) cells were incubated with indicated amount of T326 for 48 h. Cells were lysed and immunoprecipitated by anti-GFP antibodies immobilized to protein G agarose beads. After a rigorous wash, they were run on SDS-PAGE and western blotted by anti-GFP (upper panel) or anti-acetylated lysine antibodies (lower panel). *are non-specific bands.
(TIF)

Figure S4 **A:** HDAC3 inhibitor does not affect the acetylation of the proteasome. The 293T and HeLa cells were incubated with indicated HDAC inhibitors at 10×IC50, and the proteasome was extracted from sonicated cell lysates. Purified proteasomes were analyzed by western blotting using anti-20S proteasome antibody (upper panel) or anti-acetylated lysine antibodies (lower panel). The “beads” lane indicates the negative control without any cell lysates. *are non-specific bands. **B:** HDAC3 inhibitors do not affect the amount of HSP70 chaperone. Indicated amounts of HDAC3 inhibitors were added to HeLa cells and the cell lysates were analyzed by western blotting by anti-HSP70 antibody. Anti-actin blot is shown for loading control. Molecular weight markers are shown at the left side.
(TIF)

Figure S5 **HDAC3 does not bind to the proteasome.** Proteasome was purified from extracts of HeLa cells sonicated in PBS and analyzed with anti-HDAC3 or 20S proteasome antibody. Molecular weight markers are shown at the left side.
(TIF)

Table S1 **IC50 of HDAC3 inhibitors used in this study and previously reported studies.**
(DOCX)

Table S2 **Name of HeLa cell lines used in this study.**
(DOCX)

## References

[1] Boutell JM, Thomas P, Neal JW, Weston VJ, Duce J, et al (1999) Aberrant interactions of transcriptional repressor proteins with the Huntington’s disease gene product, huntingtin. Hum Mol Genet 8: 1647–1655. 10441327 10.1093/hmg/8.9.1647

[2] Steffan JS, Kazantsev A, Spasic-Boskovic O, Greenwald M, Zhu YZ, et al (2000) The Huntington’s disease protein interacts with p53 and CREB-binding protein and represses transcription. Proc Natl Acad Sci U S A 97: 6763–6768. 10823891 10.1073/pnas.100110097 PMC18731

[3] Shimohata T, Nakajima T, Yamada M, Uchida C, Onodera O, et al (2000) Expanded polyglutamine stretches interact with TAFII130, interfering with CREB-dependent transcription. Nat Genet 26: 29–36. 10973244 10.1038/79139

[4] Nucifora FC Jr, Sasaki M, Peters MF, Huang H, Cooper JK, et al (2001) Interference by huntingtin and atrophin-1 with cbp-mediated transcription leading to cellular toxicity. Science 291: 2423–2428. 11264541 10.1126/science.1056784

[5] Sadri-Vakili G, Cha JH (2006) Mechanisms of disease: Histone modifications in Huntington’s disease. Nat Clin Pract Neurol 2: 330–338. 16932577 10.1038/ncpneuro0199

[6] Steffan JS, Bodai L, Pallos J, Poelman M, McCampbell A, et al (2001) Histone deacetylase inhibitors arrest polyglutamine-dependent neurodegeneration in Drosophila. Nature 413: 739–743. 11607033 10.1038/35099568

[7] Ferrante RJ, Kubilus JK, Lee J, Ryu H, Beesen A, et al (2003) Histone deacetylase inhibition by sodium butyrate chemotherapy ameliorates the neurodegenerative phenotype in Huntington’s disease mice. J Neurosci 23: 9418–9427. 14561870 10.1523/JNEUROSCI.23-28-09418.2003 PMC6740577

[8] Hockly E, Richon VM, Woodman B, Smith DL, Zhou X, et al (2003) Suberoylanilide hydroxamic acid, a histone deacetylase inhibitor, ameliorates motor deficits in a mouse model of Huntington’s disease. Proc Natl Acad Sci U S A 100: 2041–2046. 12576549 10.1073/pnas.0437870100 PMC149955

[9] Gardian G, Browne SE, Choi DK, Klivenyi P, Gregorio J, et al (2005) Neuroprotective effects of phenylbutyrate in the N171-82Q transgenic mouse model of Huntington’s disease. J Biol Chem 280: 556–563. 15494404 10.1074/jbc.M410210200

[10] Pallos J, Bodai L, Lukacsovich T, Purcell JM, Steffan JS, et al (2008) Inhibition of specific HDACs and sirtuins suppresses pathogenesis in a Drosophila model of Huntington’s disease. Hum Mol Genet 17: 3767–3775. 18762557 10.1093/hmg/ddn273 PMC2581431

[11] Mielcarek M, Landles C, Weiss A, Bradaia A, Seredenina T, et al (2013) HDAC4 reduction: a novel therapeutic strategy to target cytoplasmic huntingtin and ameliorate neurodegeneration. PLoS Biol 11: e1001717. 24302884 10.1371/journal.pbio.1001717 PMC3841096

[12] Iwata A, Riley BE, Johnston JA, Kopito RR (2005) HDAC6 and microtubules are required for autophagic degradation of aggregated huntingtin. J Biol Chem 280: 40282–40292. 16192271 10.1074/jbc.M508786200

[13] Kazantsev AG, Thompson LM (2008) Therapeutic application of histone deacetylase inhibitors for central nervous system disorders. Nat Rev Drug Discov 7: 854–868. 18827828 10.1038/nrd2681

[14] Thomas EA, Coppola G, Desplats PA, Tang B, Soragni E, et al (2008) The HDAC inhibitor 4b ameliorates the disease phenotype and transcriptional abnormalities in Huntington’s disease transgenic mice. Proc Natl Acad Sci U S A 105: 15564–15569. 18829438 10.1073/pnas.0804249105 PMC2563081

[15] Hathorn T, Snyder-Keller A, Messer A (2011) Nicotinamide improves motor deficits and upregulates PGC-1alpha and BDNF gene expression in a mouse model of Huntington’s disease. Neurobiol Dis 41: 43–50. 20736066 10.1016/j.nbd.2010.08.017 PMC2996046

[16] Jia H, Pallos J, Jacques V, Lau A, Tang B, et al (2012) Histone deacetylase (HDAC) inhibitors targeting HDAC3 and HDAC1 ameliorate polyglutamine-elicited phenotypes in model systems of Huntington’s disease. Neurobiol Dis 46: 351–361. 22590724 10.1016/j.nbd.2012.01.016 PMC3528106

[17] Benn CL, Butler R, Mariner L, Nixon J, Moffitt H, et al (2009) Genetic knock-down of HDAC7 does not ameliorate disease pathogenesis in the R6/2 mouse model of Huntington’s disease. PLoS One 4: e5747. 19484127 10.1371/journal.pone.0005747 PMC2684627

[18] Bobrowska A, Paganetti P, Matthias P, Bates GP (2011) Hdac6 knock-out increases tubulin acetylation but does not modify disease progression in the R6/2 mouse model of Huntington’s disease. PLoS One 6: e20696. 21677773 10.1371/journal.pone.0020696 PMC3108987

[19] Moumne L, Campbell K, Howland D, Ouyang Y, Bates GP (2012) Genetic knock-down of HDAC3 does not modify disease-related phenotypes in a mouse model of Huntington’s disease. PLoS One 7: e31080. 22347433 10.1371/journal.pone.0031080 PMC3275566

[20] Katsuno M, Adachi H, Kume A, Li M, Nakagomi Y, et al (2002) Testosterone reduction prevents phenotypic expression in a transgenic mouse model of spinal and bulbar muscular atrophy. Neuron 35: 843–854. 12372280 10.1016/s0896-6273(02)00834-6

[21] Klement IA, Skinner PJ, Kaytor MD, Yi H, Hersch SM, et al (1998) Ataxin-1 nuclear localization and aggregation: role in polyglutamine-induced disease in SCA1 transgenic mice. Cell 95: 41–53. 9778246 10.1016/s0092-8674(00)81781-x

[22] Iwata A, Christianson JC, Bucci M, Ellerby LM, Nukina N, et al (2005) Increased susceptibility of cytoplasmic over nuclear polyglutamine aggregates to autophagic degradation. Proc Natl Acad Sci U S A 102: 13135–13140. 16141322 10.1073/pnas.0505801102 PMC1201602

[23] Iwata A, Nagashima Y, Matsumoto L, Suzuki T, Yamanaka T, et al (2009) Intranuclear degradation of polyglutamine aggregates by the ubiquitin-proteasome system. J Biol Chem 284: 9796–9803. 19218238 10.1074/jbc.M809739200 PMC2665101

[24] Suzuki T, Kasuya Y, Itoh Y, Ota Y, Zhan P, et al (2013) Identification of highly selective and potent histone deacetylase 3 inhibitors using click chemistry-based combinatorial fragment assembly. PLoS One 8: e68669. 23874714 10.1371/journal.pone.0068669 PMC3713009

[25] Bardai FH, Verma P, Smith C, Rawat V, Wang L, et al (2013) Disassociation of histone deacetylase-3 from normal huntingtin underlies mutant huntingtin neurotoxicity. J Neurosci 33: 11833–11838. 23864673 10.1523/JNEUROSCI.5831-12.2013 PMC3713725

[26] Duncan CE, An MC, Papanikolaou T, Rugani C, Vitelli C, et al (2013) Histone deacetylase-3 interacts with ataxin-7 and is altered in a spinocerebellar ataxia type 7 mouse model. Mol Neurodegener 8: 42. 24160175 10.1186/1750-1326-8-42 PMC3816305

[27] Bardai FH, D’Mello SR (2011) Selective toxicity by HDAC3 in neurons: regulation by Akt and GSK3beta. J Neurosci 31: 1746–1751. 21289184 10.1523/JNEUROSCI.5704-10.2011 PMC3711464

[28] Broide RS, Redwine JM, Aftahi N, Young W, Bloom FE, et al (2007) Distribution of histone deacetylases 1–11 in the rat brain. J Mol Neurosci 31: 47–58. 17416969 10.1007/BF02686117

[29] Alenghat T, Meyers K, Mullican SE, Leitner K, Adeniji-Adele A, et al (2008) Nuclear receptor corepressor and histone deacetylase 3 govern circadian metabolic physiology. Nature 456: 997–1000. 19037247 10.1038/nature07541 PMC2742159

[30] Cho Y, Sloutsky R, Naegle KM, Cavalli V (2013) Injury-induced HDAC5 nuclear export is essential for axon regeneration. Cell 155: 894–908. 24209626 10.1016/j.cell.2013.10.004 PMC3987749

[31] Yang WM, Tsai SC, Wen YD, Fejer G, Seto E (2002) Functional domains of histone deacetylase-3. J Biol Chem 277: 9447–9454. 11779848 10.1074/jbc.M105993200

[32] Jia H, Kast RJ, Steffan JS, Thomas EA (2012) Selective histone deacetylase (HDAC) inhibition imparts beneficial effects in Huntington’s disease mice: implications for the ubiquitin-proteasomal and autophagy systems. Hum Mol Genet 21: 5280–5293. 22965876 10.1093/hmg/dds379 PMC3510756

[33] Jeong H, Then F, Melia TJ Jr, Mazzulli JR, Cui L, et al (2009) Acetylation targets mutant huntingtin to autophagosomes for degradation. Cell 137: 60–72. 19345187 10.1016/j.cell.2009.03.018 PMC2940108

[34] Bence NF, Sampat RM, Kopito RR (2001) Impairment of the ubiquitin-proteasome system by protein aggregation. Science 292: 1552–1555. 11375494 10.1126/science.292.5521.1552

[35] Hipp MS, Patel CN, Bersuker K, Riley BE, Kaiser SE, et al (2012) Indirect inhibition of 26S proteasome activity in a cellular model of Huntington’s disease. J Cell Biol 196: 573–587. 22371559 10.1083/jcb.201110093 PMC3307690

[36] Bennett EJ, Bence NF, Jayakumar R, Kopito RR (2005) Global impairment of the ubiquitin-proteasome system by nuclear or cytoplasmic protein aggregates precedes inclusion body formation. Mol Cell 17: 351–365. 15694337 10.1016/j.molcel.2004.12.021

