# Supplementary material for: Differential Effect of HDAC3 on Cytoplasmic and Nuclear Huntingtin Aggregates
Source: PLoS One. 2014 Nov 7;9(11):e111277. doi: 10.1371/journal.pone.0111277 (PMC4224383; doi:10.1371/journal.pone.0111277)
Supplement: Table S1 — IC50 of HDAC3 inhibitors used in this study and previously reported studies. (DOCX) [file pone.0111277.s006.docx]

## **Table S1**

| IC50 (μM) | Class I | | | | Class IIA | Class IIB |
| --- | --- | --- | --- | --- | --- | --- |
|  | HDAC3 | HDAC1 | HDAC2 | HDAC8 | HDAC4 | HDAC6 |
| T247 | 0.24 | 19 |  | >100 | >100 | >100 |
| T326 | 0.26 | >100 |  | >100 | >100 | >100 |
| T130 | 0.57 | >100 |  | >100 |  |  |
| 138 ^16^ | 0.4 | 5.2 | 3 | 13.2 | >180 |  |
| TSA ^35^ | 0.02 | 0.006 |  |  | 0.038 | 0.0086 |
| SAHA ^35^ | 0.2 | 0.17 | 0.39 | 0.3 | 0.28 |  |
