# Supplementary material for: Differential Effect of HDAC3 on Cytoplasmic and Nuclear Huntingtin Aggregates
Source: PLoS One. 2014 Nov 7;9(11):e111277. doi: 10.1371/journal.pone.0111277 (PMC4224383; doi:10.1371/journal.pone.0111277)
Supplement: Table S2 — Name of HeLa cell lines used in this study. (DOCX) [file pone.0111277.s007.docx]

## **Table S2**

| Cell line name | #Q | C-terminal signal sequence |
| --- | --- | --- |
| C1 | 25 | none |
| C2 | 47 |  |
| C3 | 72 |  |
| C4 | 97 |  |
| E1 | 25 | NES: LALKLAGLDIN |
| E2 | 47 |  |
| E3 | 72 |  |
| N1 | 25 | NLS: DPKKKRKVDPKKKRKVDPKKKRKV |
| N2 | 47 |  |
| N3 | 72 |  |
